# XIST expression and hypermethylation of the X chromosome in males with systemic lupus erythematosus

**DOI:** 10.3389/fimmu.2026.1743606

**Published:** 2026-04-06

**Authors:** Helen O. Masson, Jonathan D. Crawford, David C. Gemperline, James E. Scherschel, Guilherme G. V Rocha, Christoph Preuss, Isabella H. Wulur, Matthew D. Linnik, Richard E. Higgs, Ernst R. Dow

**Affiliations:** 1 Eli Lilly and Company, Indianapolis, IN, United States; 2 Indiana Biosciences Research Institute, Indianapolis, IN, United States

**Keywords:** multi-omic, sexual dimorphism, systemic lupus erythematosus (SLE), X-chromosome inactivation (XCI), XIST

## Abstract

**Introduction:**

Systemic Lupus Erythematosus (SLE) exhibits a pronounced sex bias, affecting females approximately nine times more frequently than males; however, males tend to experience a more severe clinical course yet the molecular basis for these differences remains unclear.

**Methods:**

Leveraging epigenomic, transcriptomic, and proteomic data from the whole blood of 720 SLE patients (679 females, 41males) and 84 healthy controls (77 females, 7 males), we conducted comprehensive multi-omic analyses to identify sex-specific molecular features of this disease. Specifically, differential expression analysis for each modality was conducted using a factorial design to identify differences between disease and healthy controls (SLE–HC) for each sex, as well as the interaction effects between sex and disease ([Male SLE – Male HC] – [Female SLE – Female HC]). Benjamini & Hochberg false discovery rate (FDR) was used for multiple test correction.

**Results:**

The strongest signal differentiating males and females with SLE was the aberrant expression of the long non-coding RNA, *XIST*, in males. This *XIST* expression in males with SLE was bimodal, with 54% of males having elevated *XIST* expression, and correlated with disease severity. Males with SLE also exhibited significant hypermethylation of the X chromosome and transcriptional silencing of X-linked genes – hallmarks of X-chromosome inactivation (XCI), a process typically restricted to females.

**Conclusion:**

These results suggest that X-chromosome silencing by *XIST* may contribute to SLE disease in males.

## 1 Introduction

Systemic lupus erythematosus (SLE) is a systemic autoimmune disease with a striking female-bias (9:1) (1, 2), but a more severe clinical course in males (3, 4). Although the mechanisms driving this bias remains unclear, X-chromosome dosage and X-linked immune genes have been strongly implicated in SLE pathogenesis. For example, males with Klinefelter’s syndrome (XXY) have a 14-fold increased prevalence of SLE compared to XY males, while XO females have lower risk of developing SLE (2). Genome-wide association studies have identified specific X-linked genes such as *TASL* (*CXorf21*) (2, 5) and *IRAK1* (6) associated with increased SLE risk, while gain-of-function mutations in the X-linked gene *TLR7* (7, 8) have been linked to overactive toll-like receptor signaling in SLE. Another X-linked gene *XIST*, which equalizes X-gene dosage between sexes by modulating the epigenetic silencing of one of the two X chromosomes in women (9), has been associated with SLE both for its failure to completely inactivate immune genes which partially escape X-inactivation, like *TLR 7* (10), and for its immunostimulatory capacity as a long diuridine-rich RNA (11). Despite these genetic and epigenetic insights, the biological mechanisms underlying the striking sex disparity in SLE incidence and severity remains incompletely understood.

These gaps in understanding SLE heterogeneity inhibit the development of new treatments. Therefore, broader and deeper approaches to understanding the molecular biology of this complex disease are needed. Transcriptomic analyses in SLE have consistently revealed a common hallmark of disease pathogenesis: the interferon (IFN) signature (12–16). Today, methylomic and proteomic approaches have been employed to search for biomarkers, predict response to therapy, and increase our understanding of disease pathogenesis (17–23). Specifically, combined methylation and expression data on IFN-responsive genes has been shown to be more sensitive than individual components as a biomarker, while complement components and cytokines have shown utility for diagnosis and subsetting in proteomics studies (17–23). In this study, we apply multi-omic analyses – including epigenetic, transcriptomic, and proteomic profiling – on 720 SLE patients (679 females, 41 males) and 84 healthy controls (77 females, 7 males) to uncover sex-specific molecular signatures.

We present details of the sexually dimorphic signatures of SLE in patients enrolled in the BRAVE-I (NCT03616912) (24) and BRAVE-II (NCT03616964) (25) clinical trials. Notably, SLE males exhibit a bimodal distribution of *XIST* expression (22 XIST-high, 19 XIST-low), accompanied by widespread X-chromosome hypermethylation and transcriptional silencing of X-linked genes. These features of X-chromosome inactivation (XCI) were present irrespective of *XIST* expression levels. We further explore molecular and clinical differences between XIST-high and XIST-low males, showing that XIST-high males may have more severe disease. Taken together, our data highlights sex-based differences in SLE manifestation, guiding further research on the role of *XIST* and X-inactivation in SLE.

## 2 Materials and methods

### 2.1 SLE patient cohort

Our cohort consisted of the subset of patients from the BRAVE-I (NCT03616912) (24) and BRAVE-II (NCT03616964) (25) clinical trials who had given consent for all 3 omics testing (epigenomic, transcriptomic, and proteomic). This included 312 patients (293 females, 19 males) from BRAVE-I and 408 patients (386 females, 22 males) from BRAVE-II, all aged 18 years or older, with active SLE receiving stable background therapy. As previously described, these patients had a clinical diagnosis of SLE at least 24 weeks before screening; met at least 4 of 11 revised American College of Rheumatology (ACR) criteria for classification of SLE; were positive for at least one of antinuclear antibody, anti-dsDNA, or anti-Smith; had a total Systemic Lupus Erythematosus Disease Activity Index 2000 (SLEDAI-2K) score of at least 6 at screening and a clinical SLEDAI-2K score of at least 4 at baseline; and had at least one British Isles Lupus Assessment Group (BILAG) A score or two BILAG B scores at screening. The study also included 84 age-and sex-matched healthy controls – 77 females and 7 males. Serum samples (for proteomics) and whole blood samples (for transcriptomics and epigenomics) were collected at the same time in the same blood draw for each participant.

### 2.2 Proteomics

Baseline SLE serum samples were collected according to the clinical trial protocols. Blood was collected using a tube containing a clotting agent (BD SST 5ml tube). Circulating proteins were assayed using the Olink Explore 3072 panel (https://olink.com/products/olink-explore-3072-384), a proteomics platform that combines an antibody-based immunoassay with a proximity oligonucleotide extension assay and signal detection with next-generation sequencing on the NovaSeq6000 instrument (Ilumina Inc.). All serum samples were run as one batch without bridging samples, and each sample was assayed in singular. A set of internal controls (incubation, extension and amplification) was used to assess overall assay quality. Internal plate controls were used to normalize the data and to calculate the limit of detection (LOD) for each assay. Protein expression levels are given as normalized protein expression (NPX) values, which are arbitrary units used on a log2 scale that were normalized to the plate controls. For further quality control, we applied the following stringent steps to account for the observed technical variation in a subset of Olink assays that resulted in dropouts: 1) only data flagged as “PASS” according to the manufacturer’s standards passing technical QC were included; 2) only analytes with NPX values above the LOD in more than 75% of samples were included. This resulted in a total of 2,434 unique protein assays after QC that were used to explore potential novel protein markers and biological pathways in patients with SLE. Four SLE samples did not meet QC standards due to overall low expression patterns (greater than 4 standard deviations) across all assays and were excluded from the analysis.

### 2.3 RNA and DNA extraction

Whole blood RNA and DNA was extracted from PAXgene blood RNA tubes. Briefly, frozen tubes were thawed at room temperature and centrifuged according to the manufacturer’s protocol. The resulting pellet was then washed with nuclease free water, resuspended in sterile PBS, and split in half. RNA was extracted from one half of the suspension using the QIAsymphony PAXgene Blood RNA Kit (Qiagen). The other half of the suspension was used for DNA extraction using the Mag-Bind Blood & Tissue DNA HDQ Kit (Omega Bio-Tek) on the Kingfisher Flex (Thermo) instrument. RNA concentration and integrity were assessed using the Ribogreen Assay (Thermo) and a Fragment Analyzer (Agilent), respectively. The concentration and integrity for DNA was evaluated by Picogreen Assay and Agarose Gel electrophoresis.

### 2.4 RNAseq

Polyadenylated RNA was enriched using NEBNext Magnetic Oligo(dT)25 Beads and prepared into RNA-seq libraries using the NEBNext mRNA Library Prep Reagent Set for Illumina (New England BioLabs, Ipswich, MA, USA). Unique dual-index barcodes were incorporated as specified per manufacturer’s protocol. Library concentration was measured using the Quant-iT PicoGreen dsDNA Reagent (Thermo), and library size distribution and quality were checked using a DNA chip on Caliper Gx Touch (Revity). Library quantification was further refined by qPCR using the KAPA Library Quantification Kit (Roche). Paired-end sequencing (2 x 100 bp, 100 million reads per sample) was carried out on an Illumina NovaSeq 6000 platform (Illumina) following manufacturer’s recommended protocols.

Data was processed using nf-core/rnaseq v3.14.0 of the nf-core collection of workflows (26), utilizing reproducible software environments from the Bioconda (27) and Biocontainers (28) projects. The pipeline was executed with Nextflow v23.10.1 (29). Resulting count data was pre-processed using the edgeR package (30) in R, which included filtering out lowly-expressed genes using edgeR filterByExpr (min.count = 10) and performing quantile-normalized voom transformation.

### 2.5 Enzymatic methylation

Whole genome methylation libraries were constructed using 200ng gDNA input with NEBNext^®^ Enzymatic Methyl-seq Kit as per manufacturer instruction with minor modifications for automation. Library concentration was measured using the Quant-iT™ PicoGreen^®^ dsDNA Reagent (Thermo), and library size distribution and quality were checked using a DNA chip on Caliper Gx Touch (Revity). Library quantification was further refined by qPCR using the KAPA Library Quantification Kit (Roche). Paired-end sequencing (2 × 150 bp, approximately 30X coverage per sample) was carried out on an Illumina NovaSeq 6000 Plus platform (Illumina) following the manufacturer’s recommended protocols.

Data analysis was performed using nf-core/methylseq version 2.3.0 using the Bismark aligner with the GRCh38 human reference genome (GCA_000001405.15_GRCh38_no_alt fasta file with the hs38d1 decoy assembly). Most samples were deemed high quality with sufficient genome coverage > 20X. One sample was rejected as the sequencing data did not show an expected reduction in GC content observed in all other samples after sequencing, still averaging the pre-converted GC percentage of ~ 40%. The expected reduced GC content likely reflected the failure to enzymatically convert free cysteines (31, 32). Decoy sequences were padded with NN to enable extraction of relevant methylation contexts (CG, CHH vs CHG). EMseq specific trimming was set with the flag *em_seq = true*. Methylation coverage files from Bismark were imported and individual samples smoothed in R using bsseq BSmooth as the sample size was too large to jointly smooth across all samples. A gtf file was provided to summarize the smoothed methylation signals using the bsseq getMeth command resulting in a summarized methylation signal across the regions provided. Gene promotors were calculated with the GenomicRanges promoters command using default parameters for 2000 bp upstream and 200 bp downstream of TSS. Individual samples were aggregated into two methylation matrices representing gene and promotor methylation separately.

### 2.6 Chromosome read depth analysis

Mean coverage per chromosome was calculated for each patient using samtools (33), and average read depths were compared across sexes. As expected, females exhibited approximately twice the read depth for the X chromosome relative to males, while autosomal coverage was comparable between sexes (**Supplementary Figure 1**). Based on this approach, XXY Klinefelter’s males would be expected to show elevated X-chromosome coverage, comparable to females. Two outliers were identified: one male with abnormally high X chromosome read depth and one female with unusually low X-chromosome read depth. Both samples were excluded from all analyses.

### 2.7 RNA extraction & qRT-PCR

Total RNA was extracted and purified from whole blood collected in PAXgene tubes following manufacturer’s instructions. RNA concentration was determined by NanoDrop Spectrophotometer. First-strand cDNA was synthesized using SuperScript VILO cDNA Synthesis kit (Invitrogen) according to the manufacturer’s protocol. Then qRT-PCR was performed on QuantStudio 7 Flex Real-Time PCR System (Applied Biosystems) using TaqMan Fast Advanced Master Mix (Invitrogen) at 60 °C annealing temperature. Taqman assays used (XIST Hs01079824_m1, TSIX Hs03299334_s1, GAPDH Hs99999905_m1) were all obtained from Invitrogen. *XIST* and *TSIX* gene expression were normalized to *GAPDH* housekeeping gene expression in all samples analyzed in qRT-PCR.

### 2.8 Differential expression, hypergeometric, and gene set enrichment analysis

Differential expression analysis for each modality was conducted using limma (34) in R. A factorial design was applied to identify differences between disease and healthy controls (SLE-HC) for each sex, as well as the interaction effects between sex and disease ([Male SLE – Male HC] – [Female SLE – Female HC]). Benjamini & Hochberg false discovery rate (FDR) was used for multiple test correction, and hits with FDR ≤ 0.1 and |Fold Change (FC)| > 1.5 were considered significant. Genes identified as significantly differentially expressed in both male and female SLE patients from the RNAseq analysis were subjected to hypergeometric overrepresentation analysis using the enrichPathway function from the clusterProfiler (35) R package, which uses pathway terms from Reactome (36). For the other modalities (Olink and EMseq), genes were ranked by log fold change (LFC), and gene set enrichment analysis (GSEA) was performed using the gsePathway function from clusterProfiler. Significantly enriched pathways were defined by an adjusted p-value (FDR) ≤ 0.1.

### 2.9 Classification of male SLE patients based on *XIST* expression

To evaluate the potential bimodal nature of *XIST* expression in individuals with SLE, Hartigans’ dip test for unimodality was applied to both male and female *XIST* expression using the diptest package in R. Males with SLE were then systematically classified into XIST-high and XIST-low groups using kernel density estimates of qPCR-based *XIST* expression using the density function in R with the “SJ” parameter which implements the methods of Sheather & Jones (37) to select the bandwidth using pilot estimation of derivatives. The global minimum (trough) of the distribution was used as a threshold to define the two groups (**Supplementary Figure 2A**). Three male patients were missing qPCR-based *XIST* quantification. For these individuals, classification was instead based on *XIST* expression levels obtained from RNAseq data. Comparative visualization of the XIST-groupings using both qPCR and RNAseq *XIST* expression data revealed clear separation between XIST-high and XIST-low groups (**Supplementary Figure 2B**), supporting the robustness of the classification.

### 2.10 Molecular comparison of XIST-high and XIST-low males

Differential expression analysis was conducted using limma (34) in R to compare: i) XIST-high vs. healthy control males, ii) XIST-low vs. healthy control males, and iii) XIST-high vs. XIST-low males across all modalities – EMseq, RNAseq, and Olink. Multiple test correction was applied using Benjamini & Hochberg FDR, with significant hits defined by an adjusted p-value (FDR) ≤ 0.1 and |FC| > 1.5.

### 2.11 X-chromosome silencing

We constructed a custom gene set that maps each gene to their respective chromosome. Genes in the pseudoautosomal regions (PAR) were relabeled as “PAR” rather than annotated to the X- and Y- chromosomes. Using this custom gene set, we performed GSEA using the GSEA function in the clusterProfiler (35) R package comparing XIST-high, X-low, and females with SLE to their respective healthy controls across all modalities – EMseq, RNAseq, and Olink (**Supplementary Figure 3**). This approach enabled the identification of enrichments patterns at the chromosome level, rather that within predefined biological pathways. To further assess whether genes that were significantly hypermethylated (EMseq) or downregulated (RNAseq) in these subgroups were enriched in X-linked genes, we conducted hypergeometric enrichment using this custom gene set and the enricher function in the clusterProfiler (35) R package.

### 2.12 Clinical characteristics

Pairwise wilcoxon rank sum test of 95 numerical baseline clinical metrics were compared across groups: 1) XIST-high males vs females with SLE, 2) XIST-low males vs females with SLE, and 3) XIST-low vs XIST-high males with SLE. Correlations between *XIST* expression and the 95 clinical metrics was assessed using spearman correlation in R. Pairwise chi-square test were run to compare 31 categorical clinical metrics across the same 3 groups. In all cases, Benjamini & Hochberg FDR was used to correct for multiple test correction.

### 2.13 Correlation of XIST expression with type I IFN signature

Type I IFN signature was calculated by aggregating the expression of 6 type I IFN genes: MX1, OAS3, LY6E, USP18, IFI44, DDX60 (38). Spearman correlation was used to assess the association between XIST expression and the type I IFN signature in males and females separately.

### 2.14 Deconvolution of immune cell proportions from bulk RNA-seq

In-silico deconvolution of bulk RNA-seq data to estimate immune cell composition was performed using the granulator package in R (39). Raw gene counts were normalized to TPM. We evaluated 4 reference profiles (ABIS_S0-S3) (40) in combination with 7 deconvolution algorithms: dtangle (41), non-negative least squares regression model (nnls), ordinary least squares (ols), quadratic programming without constraints (qprog), quadratic programming non-negative and sun-to-one constraints (qprogwc), robust linear regression (rls), and support vector regression (svr). Notably, dtangle failed to generate cell proportions estimates for 2 of the 4 references profiles. This resulted in 26 unique deconvolution sets, which were subsequently benchmarked against immune cell proportions measured via flow cytometry (**Supplementary Figures 4A-G**). We selected the reference-method pair that achieved high concordance (Pearson correlation) with B-cell and NK-cell proportions from flow – ABIS_S0 with dtangle, resulting in the estimated composition of 17 immune cell types: naïve B-cells (B.Naive), memory B-cells (B.Memory), plasmablasts (Plasmablasts), memory helper T-cells (T.CD4.Memory), naïve helper T-cells (T.CD4.Naive), memory cytotoxic T-cells (T.CD8.memory), naïve cytotoxic T-cells (T.CD8.Naive). non-Vδ2 γδ T-cells (T.gd.non.Vd2), Vδ2 γδ T-cells (T.gd.Vd2), mucosal-associated invariant T-cells (MAIT),classical monocytes (Monocytes.C), non-classical & intermediate monocytes (Monocytes.NC.I), natural killer cells (NK), low-density neutrophils (Neutrophils.LD), low-density basophils (Basophils.LD),myeloid dendritic cells (mDCs), plasmacytoid dendritic cells (pDCs). Pairwise Wilcoxon rank sum test on the estimated cell types were compared across groups: 1) SLE females vs. female healthy controls, 2) XIST-high SLE males vs. male healthy controls, 3) XIST-low SLE males vs. male healthy controls, and 4) XIST-high vs. XIST-low SLE males. Additionally, Spearman correlations were computed between *XIST* expression levels with inferred cell type proportions.

## 3 Results

### 3.1 Patient cohort

The study cohort comprised 720 patients with active SLE, including 312 participants (293 females, 19 males) from the BRAVE-I trial and 408 participants (386 females, 22 males) from the BRAVE-II trial. All individuals were aged 18 years or older and receiving stable background therapy at the time of enrollment. Patients had a confirmed clinical diagnosis of SLE and were seropositive for at least one autoantibody—antinuclear antibody, anti-dsDNA, or anti-Smith at screening. Disease activity thresholds included a total SLEDAI-2K score ≥6 at screening, a clinical SLEDAI-2K score ≥4 at baseline, and either one BILAG A score or two BILAG B scores. Summary clinical statistics for each patient cohort are shown in **Table 1**. Additionally, 84 age- and sex-matched healthy controls were included for comparative analyses –. All participants underwent baseline sample collection for subsequent multi-omic profiling.

**Table 1:** Baseline demographics and clinical characteristics.

Clinical variable	BRAVE-I		BRAVE-II	
	F (n=293)	M (n=19)	F (n=386)	M (n=22)
Mean age, years	41.84	36.63	42.24	38.50
Mean time since onset of SLE, years	9.28	7.65	8.45	6.08
SLEDAI-2K score	9.93	9.68	10.10	10.09
CLASI score	5.67	5.79	6.98	5.41
SLICC score	0.66	0.58	0.57	0.32
PGA score	60.16	63.74	59.48	57.32
Medication				
Antimalarial	255	18	319	19
Azathioprine	49	2	61	2
Corticosteroid	211	14	318	20
Hydroxychloroquine	230	17	272	16
Immunosuppressants	173	11	210	11
Methotrexate	74	6	84	5
Non-steroidal anti-inflammatory drug	90	3	95	3
Race				
American Indian or Alaska Native	16	1	21	1
Asian	23	3	123	10
Black or African American	54	4	28	1
Multiple	1	0	6	0
White	199	11	207	10
Native Hawaiian or Other Pacific Islander	0	0	1	0

### 3.2 Proteomic, transcriptomic, and methylomic differences in SLE by sex

**Figure 1:**
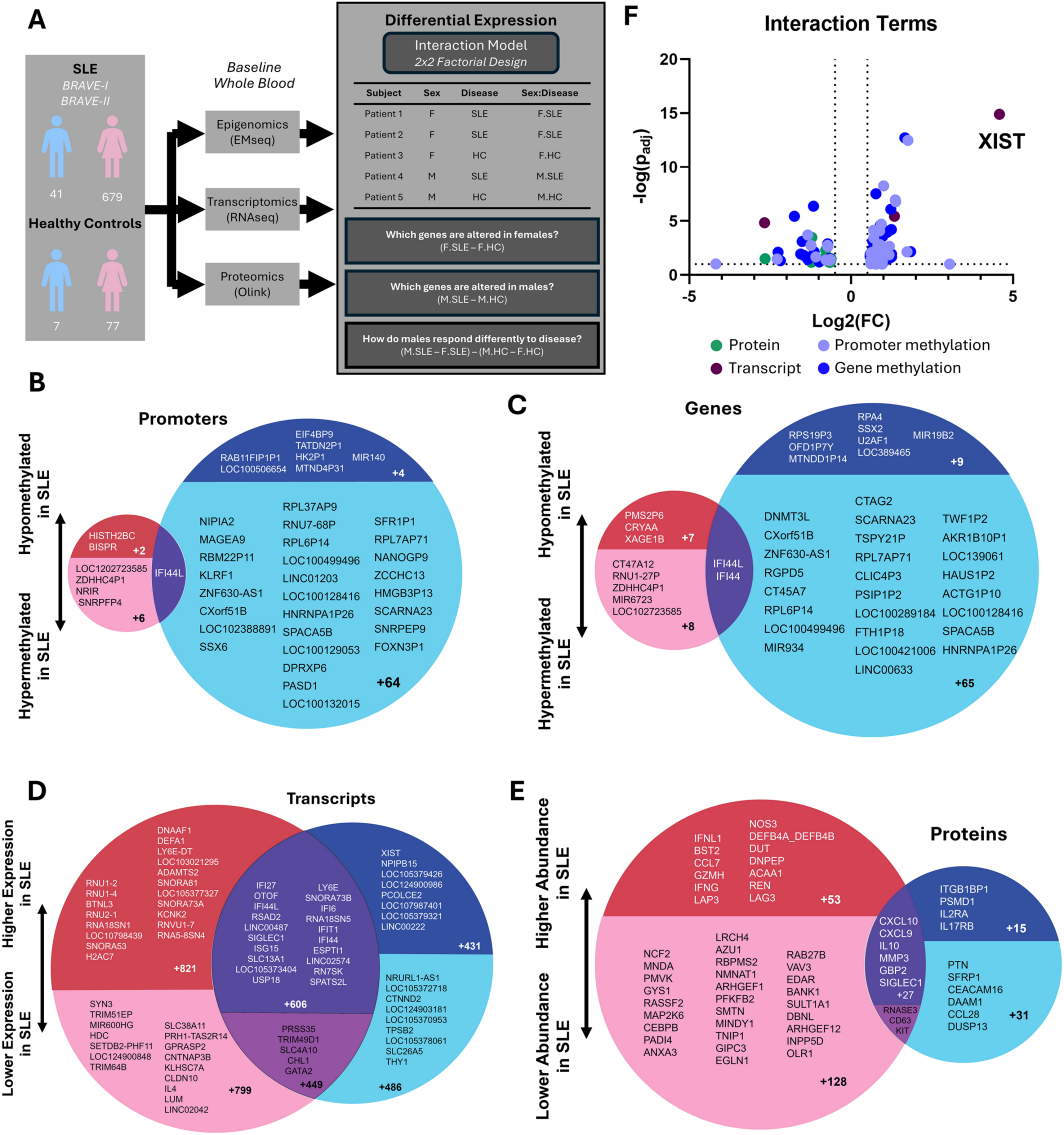
Multi-omics profiling of SLE reveals sexual dimorphism at all levels of genetic regulation. **(A)** Overview of study design. A total of 806 individuals—720 patients with SLE and 84 healthy controls—were enrolled from the BRAVE-I and BRAVE-II clinical trials. Baseline multi-omics profiling was performed using Olink proteomics, RNA sequencing (RNA-seq), and enzymatic methyl-sequencing (EM-seq). Sex-dependent molecular differences were assessed using a 2×2 factorial interaction model across each modality. **(B-E)** Venn diagrams depicting the number of significantly altered molecular features associated with disease (FDR ≤ 0.1 and |fold change| > 1.5) across four omics layers: promoter methylation **(B)**, gene methylation **(C)**, RNAseq **(D)**, and Olink **(E)**. Circle sizes are proportional to the number of significant features identified in each modality. For each Venn diagram, the left (pink) circle represents female-specific changes, and the right (blue) circle represents male-specific changes. Within each circle, upregulated features are shown in darker shades at the top, while downregulated features are shown in lighter shades at the bottom. **(F)** Volcano plot displaying all molecular features with significant sex:disease interaction effects across modalities. The x-axis represents the magnitude of the interaction effect, while the y-axis indicates statistical significance. Data points are color-coded by modality: promoter methylation (light blue), gene methylation (dark blue), Olink proteins (green), and RNA-seq transcripts (maroon).

To elucidate the molecular differences between males and females with SLE, we analyzed baseline methylome (EMseq), transcriptome (RNAseq), and proteome (Olink) data collected from whole blood from the cohort of 720 SLE patients and 84 healthy controls. Leveraging a factorial design, we performed 3 differential expression analyses for each modality: (i) SLE vs. healthy controls in females, (ii) SLE vs. healthy controls in males, and (iii) interaction between sex and disease (**Figure 1A**). The interaction analysis accounts for baseline sex differences and isolates molecular features that differ in disease response between males and females. We first focused on the sex-stratified comparisons of SLE versus healthy controls (i and ii) to characterize disease-associated molecular signatures within each sex.

#### 3.2.1 Epigenetic profiling

Males with SLE exhibited significantly more alterations in the methylome compared to females. Of the 36,175 promoter and 34,981 gene methylations quantified using EMseq, we observed 104 and 110 significant changes (|FC|>1.5, FDR<=0.1) in promoter and gene methylation relative to healthy controls, respectively, in males, compared to just 15 and 25 in females (**Figures 1B, C**). Most of these changes represented hypermethylation of DNA, associated with epigenetic silencing. Only a single promoter (*IFI44L*) and 2 genes (*IFI44L* and *IFI44*) exhibited shared methylation changes in both males and females, all of which were hypomethylated, suggesting epigenetic activation. Among these shared loci, males demonstrated a greater magnitude of change compared to females. Most epigenetic changes in males with SLE were to promoters and genes on the X chromosome (84/104 promoters and 80/110 genes). This was not the case for females, in which only 1/15 promoter changes and 3/25 gene changes were X-linked. All statistically significant changes in methylation for both sexes can be found in **Supplementary Tables 1** and **2A, B**.

GSEA analysis of the methylomes (**Supplementary Table 1** and **2C, D**) show males with SLE exhibit reduced methylation, indicative of epigenetic activation, in pathways associated with mitophagy, autophagy, protein ubiquitination, and *TNFR1* signaling compared to male healthy controls. Males with SLE also exhibited increased methylation, indicative of epigenetic silencing, in olfactory pathways dominated by olfactory receptor (OR) family genes, which are thought to be involved in modulating inflammatory macrophage activity (42, 43). Females on the other hand exhibited no significant epigenetic enrichments across any pathways, consistent with the relatively limited epigenetic changes observed in females compared to males.

#### 3.2.2 Transcriptomic profiling

Males and females with SLE exhibited comparable numbers of differentially expressed transcripts relative to healthy controls, with 2,735 in females and 2,014 in males, out of 22,443 transcripts profiled by RNAseq (**Supplementary Tables 3A, B**). Of these differentially expressed transcripts, 1,080 were shared between males and females (**Figure 1D**). Hypergeometric enrichment analysis on this set of genes commonly dysregulated in both males and females with SLE revealed that the genes upregulated in both sexes were enriched for pathways related to interferon signaling (e.g. interferon alpha/beta signaling, interferon gamma signaling, ISG15 antiviral mechanism, and regulation of INFA/INFB signaling), complement cascade (e.g. initial triggering of complement, regulation of complement cascade, and creation of C4 and C2 activators), and cell cycle/mitosis (e.g. cell cycle checkpoints, DNA replication pre-initiation, mitotic G1 phase and G1/S transition, and separation of sister chromatids). Genes commonly downregulated in both sexes showed no significant enriched pathways. A comprehensive list of enriched pathways for commonly dysregulated genes is provided in **Supplementary Tables 3C, D**.

#### 3.2.3 Proteomic profiling

Among the 2,434 proteins measured in the Olink panel, 259 showed significant changes in females (|FC|>1.5, FDR<=0.1), compared to just 92 in males (**Figure 1E**). We observed 33 proteins commonly upregulated in both sexes, including several well-characterized cytokines and chemokines. Only 3 proteins—KIT, CD63, and RNASE3—were consistently downregulated in both males and females. GSEA of the proteomes revealed shared upregulation of several immune-related pathways in both sexes, such as signaling by interleukins, cytokine signaling in immune system, and interleukin-10 signaling. In females, additional positive enrichment was observed in chemokine and interleukein-12 signaling; and negative enrichment of pathways involved in Rho GTPase signaling, tyrosine kinase signaling, phagocytosis, and death receptor signaling. The full lists of proteins with altered abundances in each sex is given in **Supplementary Tables 4A, B**, and all GSEA pathways are given in **Supplementary Tables 4C, D**.

### 3.3 *XIST* expression emerges as the dominant feature in sex-specific SLE

To investigate sex-specific molecular responses in SLE, we examined the interaction between sex and disease in our differential expression analyses. This approach identifies molecular features whose association with SLE differs between males and females, accounting for the natural differences in sex. From the EMSeq data, we identified 73 promoters and 67 genes exhibiting sex-dependent disease-associated methylation changes. Only three transcripts – *XIST*, *ZNF630*, and *NEURL1-AS1* – and 11 proteins showed significant sex-disease interaction effects (**Figure 1F**). Complete lists of promoter methylation, gene methylation, transcripts, and proteins with significant sex-disease interaction effects are provided in **Supplementary Tables 5A–D**. Notably, expression of the long non-coding RNA *XIST* emerged as the strongest and most statistically significant sex:disease interaction across all modalities. While *XIST* is a well characterized female-specific transcript with naturally variable expression between males and females, baseline sex differences have been accounted for in this model, and its persistent emergence therefore indicates an unexpected disease-specific divergence in molecular response between the sexes. In short, XIST is upregulated in SLE, but only in males.

### 3.4 Bimodal expression of *XIST* in males with SLE

**Figure 2:**
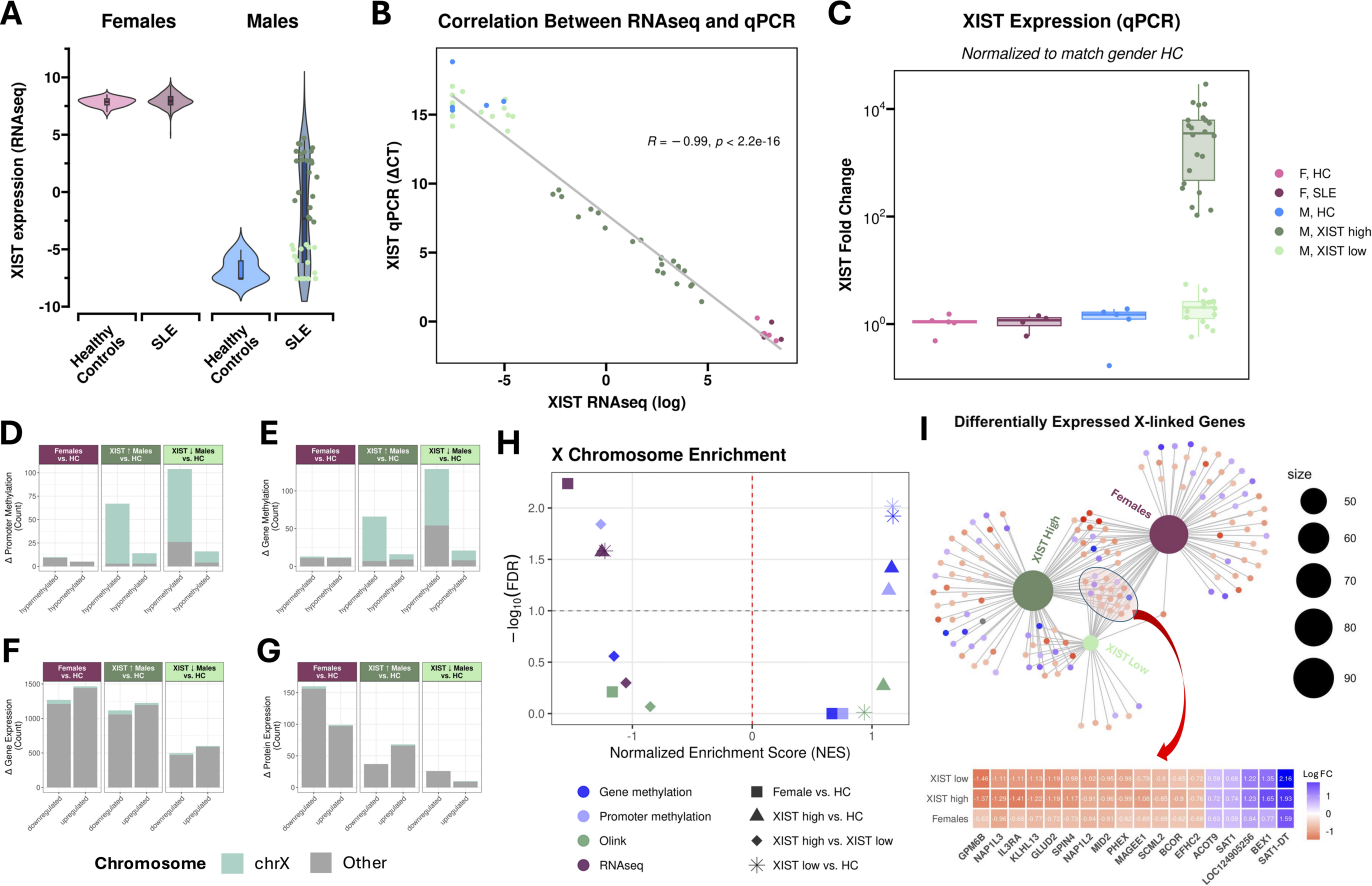
XIST expression and X-linked molecular changes in males with SLE. **(A)** Violin plots showing XIST expression as measured by RNA sequencing in females (left) and males (right) with SLE vs. healthy controls. **(B)** Scatter plot showing the correlation between XIST expression measured via RNAseq (x-axis) and qPCR (y-axis). **(C)** Boxplots of XIST expression measured via qPCR. Values here have been normalized based on expression in sex-matched healthy controls. **(D-G)** Faceted barplots illustrating the proportion of X-linked changes among all significantly hyper- and hypo-methylated promoters **(D, E)** and significantly up- and down-regulated genes and proteins **(F, G)**. **(H)** Volcano plot showing X-chromosome enrichment across the proteome (green), trancriptome (maroon), gene methylome (dark blue), and promoter methylome (light blue); the dashed grey line indicates the significance threshold (FDR ≤ 0.1). Comparisons include SLE females vs. female healthy controls (square), SLE XIST-high males vs. male healthy controls (triangles), SLE XIST-low males vs. male healthy controls (astericks), and SLE XIST-high males vs. XIST-low males (diamonds). Negtive normalized enrichment scores (NES) indicate enrichned downregulation of X-linked gene changes, reflecting X-linked silencing in the proteome and transcriptome and activation in the epigenome, and vice versa. *Scores for all other chromosomes can be found in* **Supplementary Table 6**. **(I)** Cnetplot of differentially expressed X-linked genes in females, XIST-high males, and XIST-low males compared to healthy controls. Genes commonly dysregulated across all groups are highlighted and shown in the accompanying heatmap. Note: XIST was excluded from the plot to enhance visualization due to its dominant signal.

Focusing on the strongest molecular signal differentiating males and females with SLE – *XIST* expression – we observed that females with SLE showed no significant changes in *XIST* expression relative to healthy female controls (LFC = 0.12, FDR = 0.44), while males with SLE exhibited a dramatic upregulation compared to healthy male controls (LFC = 4.71, FDR = 3.9e-18; **Figure 2A**). Further visualization of XIST expression in males revealed a bimodal distribution. To confirm this, we applied Hartigans’ dip test for unimodality to *XIST* expression in males with SLE. This yielded a p-value of 0.0027, indicating a statistically significant deviation from unimodality, consistent with a bimodal distribution of *XIST* expression in males. Analysis of *XIST* expression in females with SLE resulted in a dip test p-value of 0.99, suggesting *XIST* expression is unimodal in women.

To rule out any assay-related artifacts that could be underlying the unexpected *XIST* expression in males, we validated these findings via RT-PCR for *XIST* using all males in the cohort (41 SLE, 7 healthy controls) as well as a small set of females (4 SLE, 5 healthy controls) for comparison. The RT-PCR results closely mirrored the RNAseq data (r = 0.99, p < 2.2E-16) (**Figure 2B**), confirming the highly unusual expression of *XIST* in males with SLE. Importantly, RT-PCR confirmed the bimodal nature of *XIST* expression among males with SLE (**Figure 2C**), with a dip test p-value of 0.0008. We subsequently classified males with SLE into XIST-high (n=22) and XIST-low (n=19) phenotypes using kernel density estimates (**Table 2**).

**Table 2:** Baseline clinical demographic comparison of XIST-high and XIST-low males.

Clinical variable	XIST-high (n=22)	XIST-low (n=19)
Mean age, years	38.23	36.95
Mean time since onset of SLE, years	4.72	9.22
SLEDAI-2K score	10.68	9.00
CLASI score	5.68	5.47
SLICC score	0.41	0.47
PGA score	58.73	62.1119
Medication		
Antimalarial	19	18
Azathioprine	4	0
Corticosteroid	20	14
Hydroxychloroquine	17	16
Immunosuppressants	12	10
Methotrexate	4	7
Non-steroidal anti-inflammatory drug	4	2
Mean BILAG scores		
Constitutional	1.55	1.42
Hematological	2.27	2.21
Mucosal	4.27	3.95
Musculoskeletal	4.32	4.68
Renal	1.82	1.84

*XIST* is not canonically expressed in XY males, however it is expressed in males with Klinefelter’s syndrome (XXY), who have a 14-fold increased risk of developing SLE (44). This increased prevalence introduces a potential bias in SLE studies involving male patients. To ensure the unusual *XIST* expression was not a result of this bias, we conducted a chromosome read depth analysis to confirm accurate sex annotation and identify potential chromosomal abnormalities. This analysis revealed two individuals, one male and one female, with atypical profiles suggestive of chromosomal abnormalities (**Supplementary Figure 1**). These patients were retroactively excluded from all analyses. Importantly, their removal did not account for the observed upregulation of *XIST* expression in males.

### 3.5 Males with SLE have hypermethylated X chromosomes

Given the canonical role of *XIST* in XCI, we tested to what extent male SLE patients had hypermethylated X chromosomes, one of the silencing mechanisms of XCI (**Figures 2D, E, H**). To do this we performed a chromosomal GSEA using promoter and gene methylation comparing XIST-high males, XIST-low males, and females with SLE to their respective healthy controls. Unlike traditional GSEA which typically uses pathway or biological process level gene sets such as GO (45), KEGG (46), and Reactome (47), we created our own custom gene set that simply associated genes to their chromosomes **Supplementary Tables 6A-D**. We found that both XIST-high and XIST-low males, but not females, experienced statistically significant hypermethylation of the X-chromosome at the promoter (NES_HIGH_ = 1.14, FDR_HIGH_ = 0.06; NES_LOW_ = 1.17, FDR_LOW_ = 0.01) and gene (NES_HIGH_ = 1.16, FDR_HIGH_ = 0.04; NES_LOW_ = 1.18, FDR_LOW_ = 0.01) level in SLE compared to their sex-matched controls (**Figures 2D, E**). A complete list of all chromosomal GSEA results can be found in **Supplementary Table 6E**.

We then looked specifically at loci that were significantly hypermethylated in SLE (|FC|>1.5, FDR<=0.1) in each of these subgroups compared to their respective healthy controls. We found that in XIST-high males, 64/67 (95%) hypermethylated promoters and 59/66 (89%) hypermethylated genes were X-linked. XIST-low males showed a slightly lower prevalence, with 78/104 (75%) hypermethylated promoters and 75/129 (58%) hypermethylated genes being X-linked (**Figure 2H**). This represented statistically significant enrichments in X-linked loci among the hypermethylated promoters (FDR_HIGH_ = 2.07e-81, FDR_LOW_ = 8.18e-80) and gene (FDR_HIGH_ = 5.63e-70, FDR_LOW_ = 6.20e-64) pools in men according to a hypergeometric enrichment analysis. On the other hand, in females, only 1/10 (10%) hypermethylated promoters and 2/13 (15%) hypermethylated genes were X-linked, which did not represent a statistically significant enrichment.

### 3.6 Males and females with SLE experience transcriptional suppression of X-linked genes

In X chromosome inactivation, hypermethylation initiated by *XIST* leads to the transcriptional suppression of many X-linked genes. We therefore investigated whether male SLE patients exhibited downregulation of X-linked genes at the transcript level (**Figures 2F, H**). As with DNA methylation, we performed a chromosomal GSEA and found that all three groups (XIST-high males, XIST-low males, and females) experienced significant transcriptional suppression of the X chromosome in SLE compared to their respective healthy controls (**Figure 2H**). Unlike the epigenetic signature which showed the majority of significantly hypermethylated promoters and genes were X-linked, the proportion of X-linked genes among the pool of significantly downregulated genes was modest and comparable across groups: 60 out of 1,117 (5%) in XIST-high males, 27 out of 496 (5%) in XIST-low males, and 58 out of 1,268 (5%) in females (**Figure 2F**). None of these represented a significant enrichment in X-linked genes. In summary, the epigenetic signature in SLE males is extremely X-chromosome-enriched, but the transcriptional signature remains spread across sex and autosomal chromosomes.

To pointedly ask whether X-inactivation is occuring in these males, we examined a previously curated list of 253 transcripts known to be subject to X-inactivation for transcriptional changes in males with SLE (48). The genes considered are shown in **Supplementary Table 7**. Of these 253 XCI genes, 158 (62%) genes were downregulated (LFC < 0, FDR < 0.1) in XIST high males, while 149 (59%) were downregulated in XIST-low males compared to healthy controls. However, when we then apply our LFC threshold to look at just the pool of significantly downregulated genes (|FC|>1.5, FDR<=0.1), we find that only a small fraction of XCI genes are significantly downregulated in XIST-high males (n=22, 9%) and XIST-low males (n=7, 3%). Among the most strongly downregulated XCI genes in the XIST-high group were the lipid signaling enzyme *DGKK* (LFC = -2.23) and the RAN27A effector *SYTL5* (LFC = –1.88), while XIST-low males showed strongest downregulation of the glutamate dehydrogenase *GLUD2* (LFC = –1.19) and the histone chaperone *NAP1L2* (LFC = –1.02). These results suggest a potential link between elevated *XIST* expression in males and partial silencing of X-inactivated genes, although other chromosomes experience proportionally just as many transcriptional changes.

Given the significant transcriptional repression of the X-chromosome indicated via GSEA across all subgroups, we next focused on the set of X-linked genes significantly dysregulated in each subgroup relative to healthy controls (**Table 3**; **Figure 2I**). In XIST-high males, 90 X-linked genes were significantly dysregulated, with 60 downregulated and 30 upregulated. XIST-low males showed fewer changes, with 41 dysregulated genes (27 downregulated, 14 upregulated). Females with SLE exhibited 83 dysregulated X-linked genes, including 58 downregulated and 25 upregulated. Notably, 16 X-linked genes were differentially expressed in both XIST-high and XIST-low males but remained unchanged in females, which could indicate potential male-specific regulatory mechanisms. Additionally, 18 genes were consistently dysregulated across all three groups. Among these, *IL3RA* (CD123), which encodes the α-subunit of the interleukin-3 receptor and serves as a key marker of plasmacytoid dendritic cells (pDCs) was commonly downregulated—most prominently in XIST-high males (LFC = –1.41, FDR = 0.002), followed by XIST-low males (LFC = –1.11, FDR = 0.04), and then females (LFC = –0.68, FDR = 4.4e-11). To further assess potential pDC involvement, we examined *CLEC4C*, another canonical pDC marker, which exhibited a similar trend: downregulated strongest in XIST-high males (LFC=-1.12, p=0.03, FDR = 0.1), followed by XIST-low males (LFC=-1.0, p=0.05, FDR = 0.24), and females (LFC=-0.65, p= 4.4e-06, FDR = 1.3e-05). The full results of the differential expression analyses of XIST-high males and XIST-low males versus healthy controls are shown in **Supplementary Table 8A**.

**Table 3:** Top 20 differentially expressed X-linked genes in SLE females, XIST-high, and XIST-low males vs. sex-matched healthy controls.

Females			XIST-high Males			XIST-low Males		
Gene	LFC	FDR	Gene	LFC	FDR	Gene	LFC	FDR
LOC105373180	1.82	2.85E-15	XIST	8.47	7.58E-119	SAT1-DT	2.16	1.07E-02
CSAG3	1.63	4.81E-12	DGKK	-2.23	2.33E-02	PAGE1	2.01	2.83E-02
SAT1-DT	1.59	4.45E-22	SLC16A2	-2.18	1.12E-02	LOC107985657	-1.59	6.87E-02
GPRASP2	-1.58	1.34E-04	SAT1-DT	1.93	8.87E-03	LOC124905180	-1.58	3.82E-02
SLC16A2	-1.52	1.52E-15	LOC105373180	1.89	8.34E-02	GPM6B	-1.46	8.26E-02
LOC105373175	-1.51	3.92E-13	SYTL5	-1.88	5.34E-02	POF1B	-1.38	8.73E-02
SYTL5	-1.45	3.92E-12	LOC729609	1.87	1.77E-02	BEX1	1.35	8.66E-02
SNORA11	1.43	2.91E-06	LOC107985677	1.75	3.33E-02	ZNF630	1.27	1.10E-04
DGKK	-1.42	3.97E-11	BEX1	1.65	1.26E-02	LOC124905256	1.22	1.19E-02
GPRASP3	-1.40	7.53E-04	PAGE1	1.62	4.48E-02	GLUD2	-1.19	4.52E-02
SRPX	-1.35	5.82E-14	LOC107985657	-1.54	4.22E-02	KLHL13	-1.13	1.40E-02
NALF2	1.35	2.58E-10	SRPX	-1.52	7.29E-02	IL3RA	-1.11	4.01E-02
GK-AS1	1.14	2.95E-09	LOC107985695	-1.45	3.27E-02	NAP1L3	-1.11	6.54E-02
CASK-AS1	1.08	1.31E-17	IL3RA	-1.41	2.06E-03	ZNF157	-1.08	3.64E-02
GPC3	-1.02	1.16E-09	LOC124905239	-1.40	7.20E-02	NAP1L2	-1.02	5.47E-02
DMRTC1B	-0.99	3.15E-05	GPM6B	-1.37	6.20E-02	PHEX	-0.98	3.18E-02
LOC105377212	-0.98	1.33E-04	ZNF157	-1.36	1.70E-03	SPIN4	-0.98	1.71E-02
RPA4	0.97	6.51E-25	LOC105373177	-1.35	9.34E-02	ASMT	0.96	8.65E-02
RHOXF1P1	-0.97	1.77E-07	LOC124905180	-1.33	4.65E-02	MID2	-0.95	6.34E-02
NAP1L3	-0.96	1.68E-17	ZNF630	1.33	1.41E-06	LOC124905203	0.93	3.24E-02

### 3.7 Minimal changes in the expression of X-linked proteins in the Olink panel

Of the proteins profiled using the Olink panel, only 66 mapped to the X chromosome. Among females with SLE, 6 X-linked proteins were differentially expressed relative to healthy controls: VSIG4 and SH2D1A were upregulated, while IKBKG, CD40LG, NAA10, and ITGB1P2 were downregulated. In males, both XIST-high and XIST-low groups exhibited differential expression of only 2 X-linked proteins relative to healthy male controls, each. VSIG4 was consistently upregulated in both groups, while RP2 was additionally upregulated in XIST-high males, and DIPK2B was downregulated in XIST-low males (**Supplementary Table 8B**). Unlike with the epigenome and transcriptome, we did not observe an enrichment of X-linked protein activation or suppression in any of the groups (**Figures 2G, H**). This lack of apparent protein-level suppression may be partially attributable to the limited genomic coverage and inherent bias of the Olink panel.

### 3.8 Shifts in XIST expression in males does not reflect changes in cell type composition

**Figure 3:**
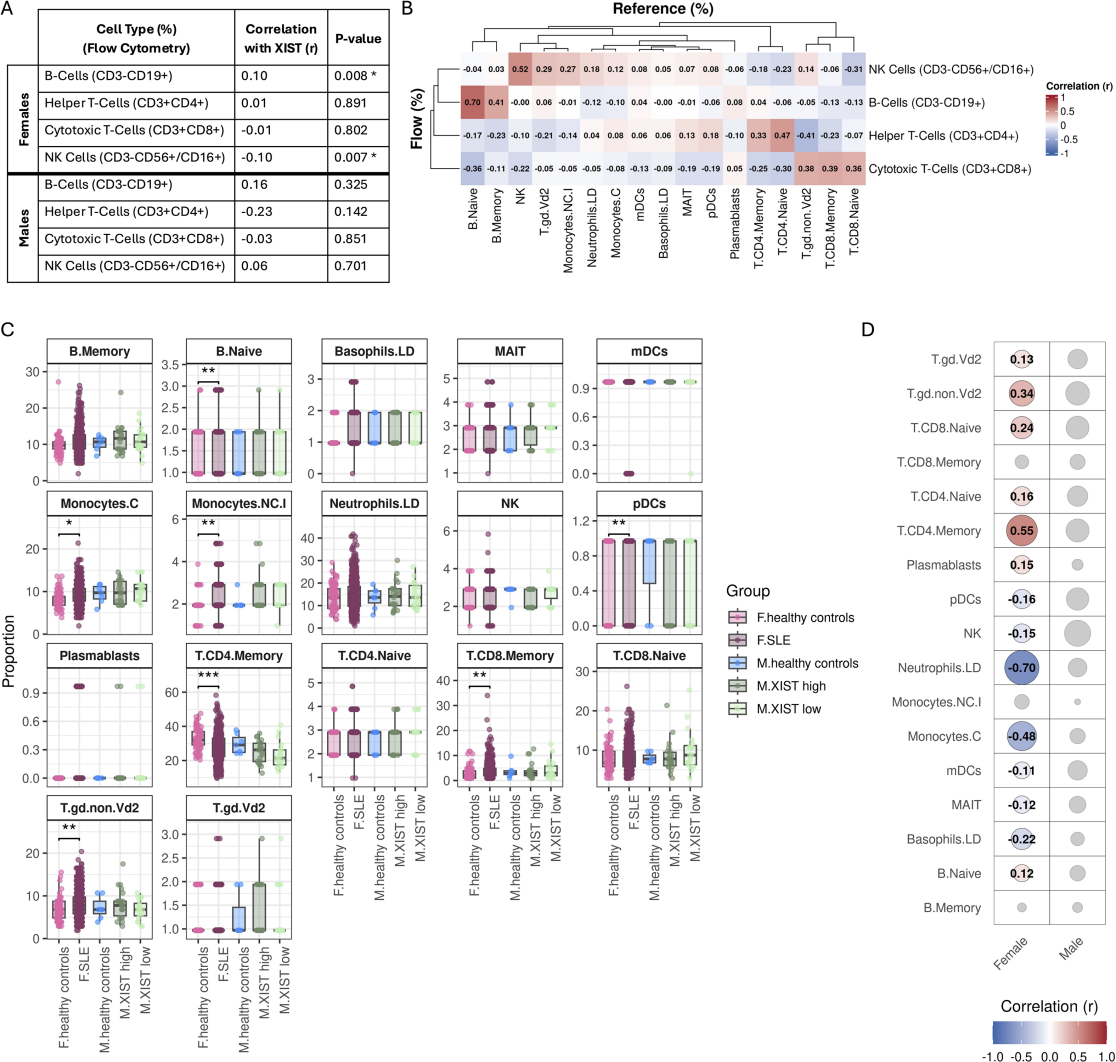
XIST Expression is uncoupled from immune cell composition in SLE male cohorts. **(A)** Correlations between XIST expression levels and major immune cell types measured in our female (top) and male (bottom) SLE patient cohort, measured by flow cytometry. Significant correlations (p-value ≤ 0.01) are marked with an asterisk. **(B)** Benchmark correlation matrix of the top-performing reference panel used in cell-type deconvolution. This panel (y-axis), comprising 17 immune cell types, showed strong correlation with B-cells from the clinical flow panel (x-axis), while maintiaing one of the strongest correlations with NK cells. **(C)** Deconvoluted cell type proportions for each group: female healthy controls, females with SLE, male healthy controls, XIST high males with SLE, and XIST low males with SLE. Significant differences (p ≤ 0.01) between SLE subgroups and sex-matched healthy controls are highlighted: *0.001-0.01, **0.0001-0.001. **(D)** Correlations between deconvoluted immune cell types and XIST expression in males (top) and females (bottom) in our SLE patient cohort. Dot color and size indicate correlation strength (r) and significance (p-value), respectively. Non-significant correlations are shown in gray.

Bulk whole-blood sequencing captures a mixture of numerous immune cell types, each with different epigenetic landscapes, activation states, and expression profiles. To assess whether differences *XIST* expression reflect shifts in immune cell composition within the patient cohort, we examined correlations between *XIST* levels and the proportions of major immune subsets measured by flow cytometry: B-cells (CD3-CD19+), helper T-cells (CD3+CD4+), cytotoxic T-cells (CD3+CD8+), and natural killer (NK) cells (CD3-CD56+/CD56+) (**Figure 3A**). In females, *XIST* expression showed a weak but statistically significant positive association with B cell frequency, and a negative association with neutrophil frequency. These findings align with prior reports that *XIST* expression is highest in B-cells (11). In contrast, no significant correlations were observed between XIST levels and immune cell subsets in males.

While flow cytometry provided proportions for major immune cell types, measurements for several relevant subsets were missing. To obtain a more comprehensive profile of immune cell populations, we performed in silico deconvolution of the bulk RNA-seq data. We first benchmarked 4 immune cell reference profiles and 7 deconvolution algorithms against the available flow cytometry measurements (**Supplementary Figure 4**). Given the established role of B-cells in SLE, we selected the reference/method combination that showed high concordance with B-cell proportions while also maintaining good agreement with NK proportions as measured by flow cytometry (**Figure 3B**). This combination resulted in the inference of 17 immune cell subsets.

Comparison of immune cell subsets across SLE subgroups (females, XIST high males, and XIST low males) versus sex-matched healthy controls revealed only a few significant shifts in females and none in males (**Figure 3C**). Moreover, a direct comparison between XIST high and XIST low males showed no significant differences in any of the deconvoluted cell types, suggesting that variation in *XIST* expression does not correspond to changes in immune cell composition. However, rare subsets, such as dendritic cells and plasmablasts, are known to occur at very low levels in circulation, making accurate deconvolution challenging. Therefore, we are cautious about relying on estimates of these rare subtypes for meaningful interpretation.

We next examined correlations between these inferred cell proportions and *XIST* expression (**Figure 3D**). In females, *XIST* levels showed low-moderate positive associations with a variety of T- and B-cell, and a moderately strong negative with neutrophils (r=-0.70). We also observed weaker negative correlations with dendritic, basophil, NK, classical-monocyte, MAIT cells. In contrast, males exhibited no significant correlations with any immune cell subsets, consistent with findings from flow-based analyses. Collectively, these results suggest that the observed variation in XIST expression among males in unlikely to be driven by differences in immune cell composition.

### 3.9 Molecular differences between XIST-high and XIST-low males

**Figure 4:**
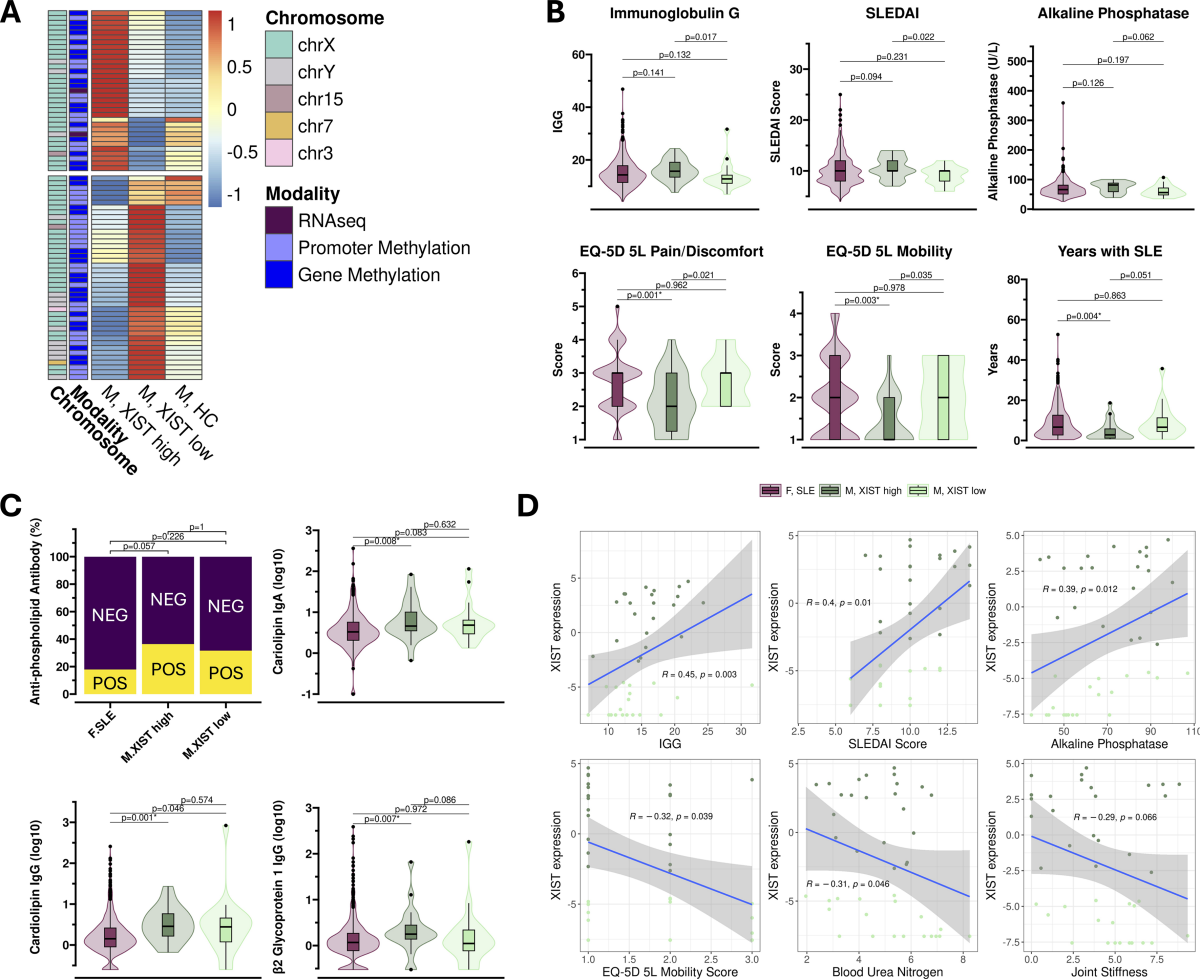
Molecular and clinical distinctions between XIST-high and XIST-low males with SLE. **(A)** Clustered heatmap of the 75 molecular differences between XIST-high and XIST-low males (FDR<= 0.1, |FC|>1.5). Colors represent mean-scaled expression across XIST-high males (left), XIST-low males (center), and male healthy controls (right). The top cluster includes features upregulated in XIST-high males, while the bottom cluster includes features downregulated expression in XIST-high males compared to XIST-low males. The majority of these differences occur in the epigenome (modality = blue) on the X-chromosome (chromosome = aqua). **(B)** Violin plots comparing clinical measurements among females (maroon), XIST high males (dark green), and XIST low males (light green) with SLE. Non-adjusted p-values from Wilcoxon rank-sum tests are displayed above each comparison. **(C)** APS-related clinical markers. Stacked barplot depicts proportion of antiphospholipid antibody status at baseline per group, and violin plots show distribution of APS-associated antibody levels among females (maroon), XIST-high males (dark green), and XIST-low males (light green) with SLE. Chi-square and Wilcoxon rank-sum test p-values are displayed above. Comparisons that remain significant after multiple-hypothesis testing (FDR ≤ 0.1) are highlighted: *0.01-0.1, **0.001-0.1. **(D)** Associations between XIST expression and select clinical parameters. Scatter plots depict correlations between XIST expression levels in XIST-high (dark green) and XIST-low (light green) males with SLE and IgG levels, SLEDAI score, alkaline phosphatase levels, EQ-5D 5L Mobility Score, urea nitrogen levels in blood, and joint stiffness. Spearman correlation coefficients (R) and associated p-values are shown to indicate strength and significance of each association.

Despite the pronounced differences in *XIST* expression, males with high and low *XIST* levels were remarkably similar at the molecular level, with only 75 significant differences (|FC|>1.5, FDR<=0.1) detected across all modalities—EM-seq, RNA-seq, and Olink (**Figure 4A**) (**Supplementary Tables 8, 9**). The majority of these differences were epigenetic, comprising 35 gene-level and 38 promoter-level changes (**Supplementary Tables 9A, B**). Most of these epigenetic alterations were sex-linked: 30 of 38 promoters and 27 of 35 genes were X-linked, while 6 promoters and 6 genes were Y-linked. Beyond *XIST*, only one transcript, *RPS4Y2*, was differentially expressed between the two groups, showing elevated expression in the XIST-high cohort. *RPS4Y2*, a Y-linked ribosomal protein gene, is typically restricted to the prostate and testis and preferentially expressed during spermatogenesis (49). Although one computational study has proposed RPS4Y2 as a potential biomarker in rheumatoid arthritis (RA) (50), no functional or mechanistic role in immune regulation has been described. Proteomic analysis revealed no significant differences between the groups.

### 3.10 Clinical correlates of XIST expression in SLE

To assess whether *XIST* expression stratifies clinical heterogeneity among males with SLE, we conducted pairwise comparisons of XIST-high, XIST-low, and female SLE subgroups across 126 clinical parameters. After correction for multiple comparisons (FDR<0.1), no individual clinical variable remained statistically significant between XIST-high to XIST-low males. However, several clinical markers showed nominal associations that may warrant further investigation. For instance, XIST-high males exhibited higher SLEDAI scores (p = 0.022, FDR = 0.70), elevated serum IgG (p = 0.017, FDR = 0.70), and increased alkaline phosphatase levels (p = 0.062, FDR = 0.87) compared to XIST-low males, potentially reflecting more active disease. Conversely, XIST-low males reported greater pain and mobility issues, as indicated by higher EQ-5D-5L Pain/Discomfort (p = 0.021, FDR = 0.70) and Mobility scores (p = 0.035, FDR = 0.83), along with longer disease duration (p = 0.051, FDR = 0.87) (**Figure 4B**).

While there were no statistically significant clinical differences between XIST-high and XIST-low males, a comparison of XIST-high and -low males to females with SLE did reveal several significant clinical differences. Both XIST-high and XIST-low males exhibited higher uric acid, serum ferritin, and creatine levels, and lower HDL cholesterol compared to women with SLE. XIST-high males showed additional differences including elevated antiphospholipid antibody profiles (**Figure 4C**), suggesting a potentially increased risk of antiphospholipid syndrome (APS) in this subgroup. These observations, while not statistically robust, may point to biologically relevant trends that merit validation in larger cohorts. A complete list of clinical variables and associated statistics is provided in **Supplementary Table 10**.

Next, we examined correlations between *XIST* expression in SLE and the subset of 95 numerical clinical parameters (**Supplementary Table 11**). When looking at males and females combined, we observed statistically significant correlations with neutrophil count (r = -0.33, FDR = 5.26e-18) and T-cell counts (r = 0.33, FDR = 5.26e-18). However, when we look at only the males, no associations reached statistical significance after multiple testing correction. The strongest correlations in males included serum IgG levels (r = 0.45, p = 0.003, FDR = 0.16), SLEDAI scores (r = 0.40, p = 0.010, FDR = 0.28), and serum alkaline phosphatase levels (r = 0.39, p = 0.012, FDR = 0.28) (**Figure 4D**), consistent with elevated values of these parameters in the XIST-high group. The most pronounced negative correlations in males included EQ-5D-5L mobility scores (r = –0.32, p = 0.039, FDR = 0.72), blood urea nitrogen (r = –0.31, p = 0.046, FDR = 0.72), and baseline joint stiffness severity (r = –0.29, p = 0.066, FDR = 0.72). Finally, given previously identified correlations between XIST expression and the IFN signature, we evaluated this correlation in male and female SLE patients. We did not observe any correlation between XIST expression and IFN in males, and observed a weak, negative correlation in females (**Supplementary Table 12**).

Taken together, these results reveal consistent trends associating elevated XIST expression in male SLE patients with increased disease activity and immunological markers, while lower XIST expression correlates with greater joint pain, impaired mobility, and indicators of renal dysfunction—suggesting that XIST levels may delineate clinically distinct subgroups within male SLE patients.

## 4 Discussion

Herein we report a multi-omic analysis of epigenetic, transcriptomic, proteomic and clinical data on baseline samples collected from over 700 SLE patients enrolled in two phase 3 clinical trials. Our work gives a detailed account of the sexually dimorphic epigenetic, transcriptomic, and proteomic response in SLE. Our multi-omics approach allowed us to observe a more complete picture of how changes at the epigenetic level relate to those at the transcriptional, proteomic, and clinical levels.

Accounting for baseline molecular differences between males and females, we identified elevated expression of the long non-coding RNA *XIST* in males with SLE as the most prominent signal distinguishing sex-specific associations with disease across diverse multi-omic profiles. While *XIST* expression is a well-established feature of female biology due to its role in XCI, its somatic expression in males is highly atypical. Upon further investigation, the expression of *XIST* is males was bimodal with approximately half expressing significantly higher levels of *XIST* compared to male healthy controls. Consistent with *XIST* expression, males with SLE experience significant hypermethylation of the X chromosome, a hallmark of XCI. Notably, this epigenetic silencing is observed not only in XIST-high individuals but also in those with low XIST expression comparable to healthy controls. Given that *XIST* RNA mediates XCI through hypermethylation (9, 51), we hypothesized that the hypermethylation observed in males with SLE may result from *XIST* upregulation, potentially leading to a form of X-inactivation in male immune cells. Supporting this, we found that males with SLE exhibit reduced overall transcription from the X chromosome compared to healthy controls, although only ~5% of downregulated transcripts in male SLE are X-linked. Moreover, the pattern of gene silencing in SLE males appears distinct from canonical female X-inactivation. Specifically, when analyzing a curated set of known XCI targets (48), we observed that most (> 50% in both XIST-high and XIST-low males) were downregulated (LFC < 0, FDR < 0.1) but not totally inactivated (|FC| < 1.5). These findings suggest aberrant XIST expression in males with SLE, potentially leading to transcriptional silencing of some X-linked genes or contributing to SLE pathogenesis in other ways.

Importantly, the correlation of XIST expression with male SLE does not imply causation, and mechanistic follow-up is warranted to determine whether XIST upregulation drives pathology or is a consequence of immune activation in SLE. Upregulation of *XIST* in males has been implicated in a handful of instances of extreme genetic dysregulation, such as somatic cancers (52, 53) and in pulmonary arterial hypertension (54). To our knowledge, *XIST* upregulation has not previously been reported in males with SLE or other autoimmune diseases (55). Importantly, this phenomenon is not due to chromosomal abnormalities in our patients.

XIST has previously been reported to be more highly expressed in immune cells of women with SLE, correlated with SLEDAI scores, and correlated with the interferon signature in kidneys of women with lupus nephritis (11). XIST has also been identified as a binding partner for many lupus autoantigens (56), and knock-in of a non-silencing form of XIST to lupus-prone male mice induces lupus-associated autoantibodies, exacerbates organ pathology, and expands atypical B cell populations—cells known to depend on TLR7 stimulation (57–59). It has previously been hypothesized that the long, diuridine rich nature of the XIST RNA enables it to drive chronic TLR7 activation and thus IFN production (11). Reverse causation – XIST induction by IFN – has been investigated with both targeted and unbiased approaches and not been found (11, 60, 61), although this possibility has certainly not been exhausted. Indirect regulation of XIST downstream of immune activation due to transcriptional stochasticity or clonal hematopoiesis such as in cancers could be an alternative explanation (50–52, 62). Our finding that males with SLE also express XIST justifies rigorous follow-up into the causes and consequences of XIST upregulation in male immune cells.

Notably, *XIST* expression is bimodal in men with SLE. This may indicate that some men with SLE do not dramatically upregulate XIST expression, or that XIST upregulation is a transient element of SLE disease and not permanent. Notably, even XIST-low SLE men experienced a large degree of epigenetic change on the X chromosome. One possible explanation is that *XIST*-mediated DNA modifications are highly stable and persist even after *XIST* transcription diminishes (63, 64). Thus, XIST-low males may have previously expressed *XIST*, retaining the associated epigenetic marks despite reduced current expression. This bimodal expression allowed us to investigate the clinical consequences of *XIST* upregulation in males. While no clinical parameters met significance after multiple testing correction, we observed nominal associations of *XIST* expression with a higher SLEDAI score, more serum IgG, higher serum alkaline phosphatase, fewer years with SLE, lower EQ-5D 5L Pain/Mobility scores, and a higher likelihood of anti-phospholipid antibodies. Overall, these results seem to suggest a positive correlation between *XIST* expression and disease activity in the blood, and possibly a negative correlation with musculoskeletal disease activity. These results are consistent with previous reports that *XIST* expression levels in female immune cells positively correlate with SLEDAI scores (11), although our larger cohort did not replicate that report’s finding of higher XIST levels in females with SLE. The correlations between XIST and SLEDAI score and XIST and total serum IgG align with the results of XIST knock-in to murine SLE-susceptible mice (59).

Notably, *XIST* expression is highest in males with newly diagnosed SLE, suggesting that its upregulation may occur early in disease development. We do not believe this finding is due to confounding *XIST* changes with age; in fact, it contrasts with the established trend of increasing *XIST* levels with age in the general population (65, 66). It is important to note that these findings represent trends which do not survive multiple comparisons corrections for all 126 variables we analyzed. We did not observe a correlation between XIST and the IFN signature in men, and observed a weakly negative correlation in women. This contrasts with published findings in the tissue, where XIST has been positively correlated with IFN levels (11). Our finding that pDC markers IL3RA and CLEC4C are downregulated most in XIST-high males, followed by XIST-low males and then females, may suggest that XIST levels are higher in individuals with more pDCs leaving the blood to penetrate the tissue, although we do not have the granularity to assess this.

Despite the strengths of our multi-omics approach and the large cohort analyzed, several limitations warrant consideration. First, while our study provides novel insights into SLE pathophysiology in males, it is important to acknowledge that SLE predominantly affects females. Since the bimodal expression of *XIST* was only detected in male SLE patients, this limits the direct generalizability to the broader SLE population. In females, the complexity of XCI and the presence of two X chromosomes introduce additional layers of regulation that may obscure the signal observed in males. For example, although we didn’t observe a statistically significant difference in mean *XIST* expression between female SLE patients and healthy controls, we did detect a significant increase in the variability of *XIST* expression in women with SLE using Levene’s test (p = 0.0005). This suggests what while a clear bimodal signal is not evident in females at the whole blood level, it may still exist to a lesser degree or in a subset of cells which is obscured by the baseline expression of *XIST*. Thus, future studies will be essential to translate these findings to female patients and determine whether similar mechanisms are at play, albeit in a more nuanced or context-dependent manner.

Second, although this study includes one of the largest male SLE cohorts reported to date, the sample size remains a constraint, particularly when stratifying patients into XIST-high and XIST-low subgroups. This subdivision reduced statistical power and limited our ability to fully characterize the clinical and molecular differences between these groups. Additionally, the relatively small number of male healthy controls may influence the robustness of observed male-specific molecular changes. Some findings could reflect sampling bias rather than true disease-associated effects, and caution is warranted in drawing strong conclusions without further validation. Further validation in larger, independent cohorts, as well as rigorous mechanistic investigation, will be essential in confirming these observations and clarifying the biological relevance of *XIST*-associated changes in male SLE.

Third, confounding explanations for XIST upregulation could escape the limits of detection of our assays; for example low-level aneuploid mosaicism (where males have some XXY immune cells) could escape read detection for chromosomal abnormalities in bulk RNA sequencing but provide XIST reads. This hypothesis could also underlie the upregulation of XIST seen in some female cohorts, as some women may have low level mosaicism with XXX immune cells. Addressing this hypothesis requires deeper resolution and further interrogation in immune sub-populations via single-cell or flow sorted type of analyses.

Finally, methylation data alone cannot define whether these males are experiencing targeted gene regulation or something similar to X-chromosome inactivation. We generally interpret hypermethylation as gene silencing, but this is not always the case: for example, gene body methylation can sometimes lead to more active transcription rather than repression (67).

This study challenges conventional approaches to omic analysis in sexually dimorphic diseases like SLE. Historically, male patients have been underrepresented, and in some cases excluded entirely. When included, males are often analyzed separately, or sex is treated as a nuisance variable and regressed out to isolate disease differences. These approaches risk overlooking critical biological insights into the sex-based differences that share disease manifestation and progression. Here we’ve modeled sex as an interaction term, enabling comparisons between male and female SLE patients while accounting for baseline sex differences. This framework reveals novel disease-specific sex differences that are otherwise masked, like *XIST*, which may hold key insights into disease mechanisms and potential therapeutic targets.

## Data Availability

The data presented in the study are deposited in the NCBI GEO repository, accession numbers GSE318618, GSE319130 and GSE319595.
