# Supplementary material for: XIST expression and hypermethylation of the X chromosome in males with systemic lupus erythematosus
Source: Front Immunol. 2026 Apr 6;17:1743606. doi: 10.3389/fimmu.2026.1743606 (PMC13103550; doi:10.3389/fimmu.2026.1743606)
Supplement: Supplementary file 1 [file DataSheet1.docx]

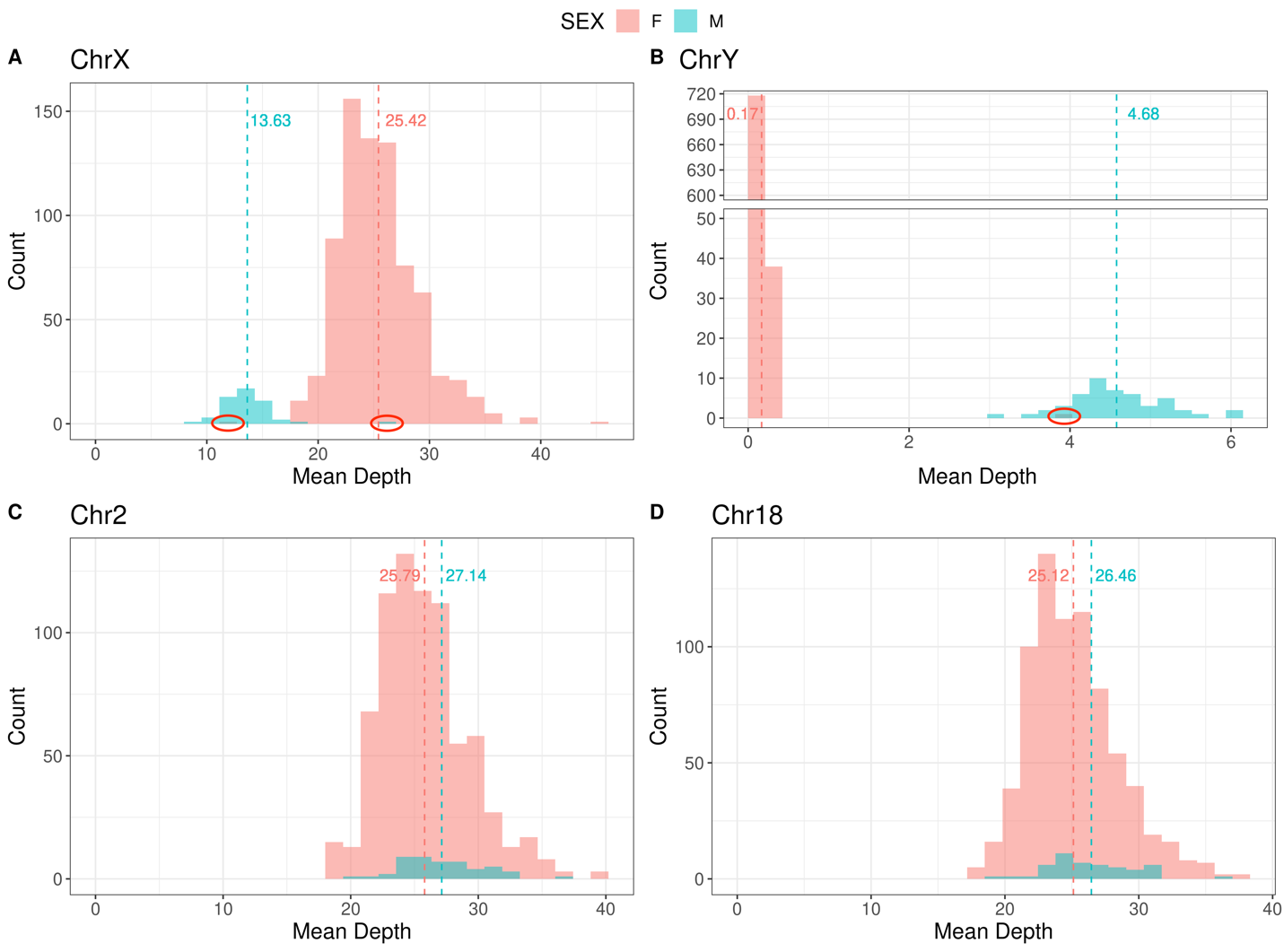


**Supplementary Fig. 1. Mean chromosome coverage depth.**  Histogram plots showing the mean depth coverage of the sex chromosomes, ChrX **(A)** and ChrY **(B)**, along with two autosomal chromosomes for comparison, Chr2 **(C)** and Chr18 **(D)**. The means for each sex are depicted with a dashed line. Clear outliers are circled in red.


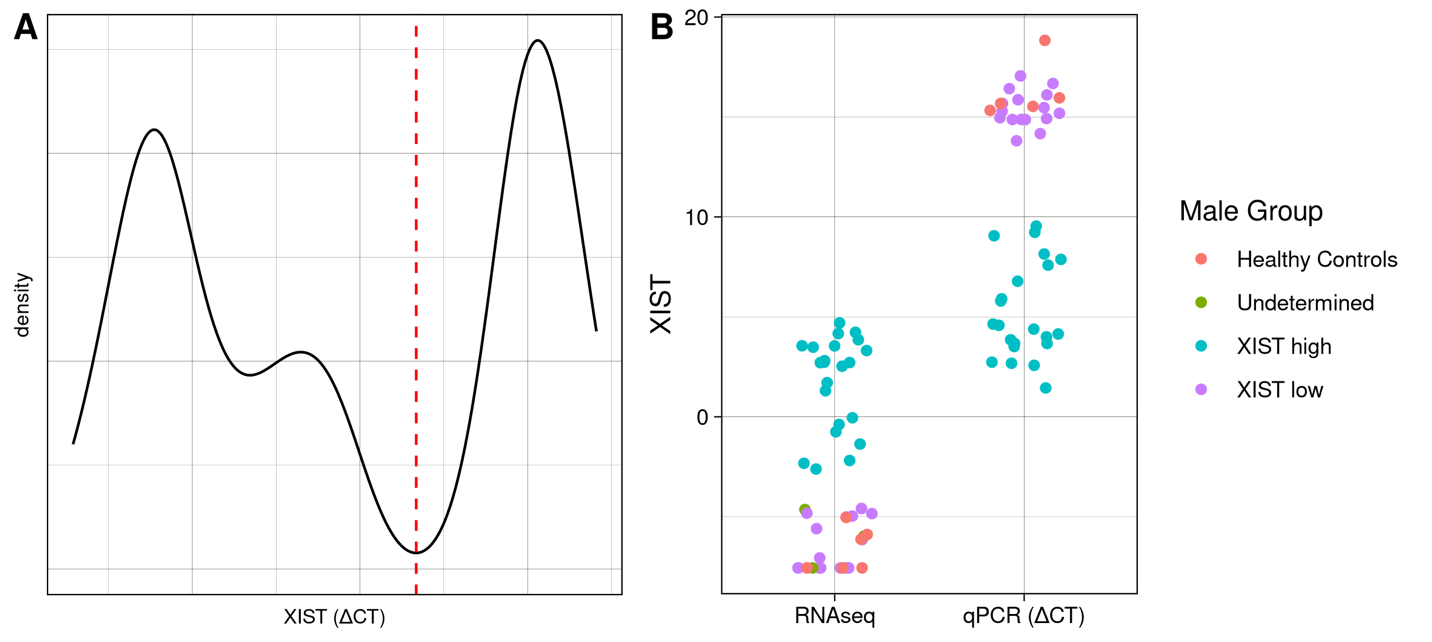


**Supplementary Figure 2. Classification of male SLE patients based on XIST expression.**  **(A)** Kernel density plot of XIST expression (ΔCT) measured by qPCR in male SLE patients. The dashed red line marks the global minimum of the distribution and was used to stratify patients into XIST-high (left of the threshold) and XIST-low (right of the threshold) groups. **(B)** Scatter plots of XIST expression measured by RNA-seq (left) and qPCR (right), with data points colored by classification: healthy controls (pink), XIST-high (blue), and XIST-low (purple). Three male patients lacking qPCR data are shown in green in the RNA-seq plot. Based on their RNA-seq expression levels and visual separation from the XIST-high group, these individuals were classified as XIST-low.


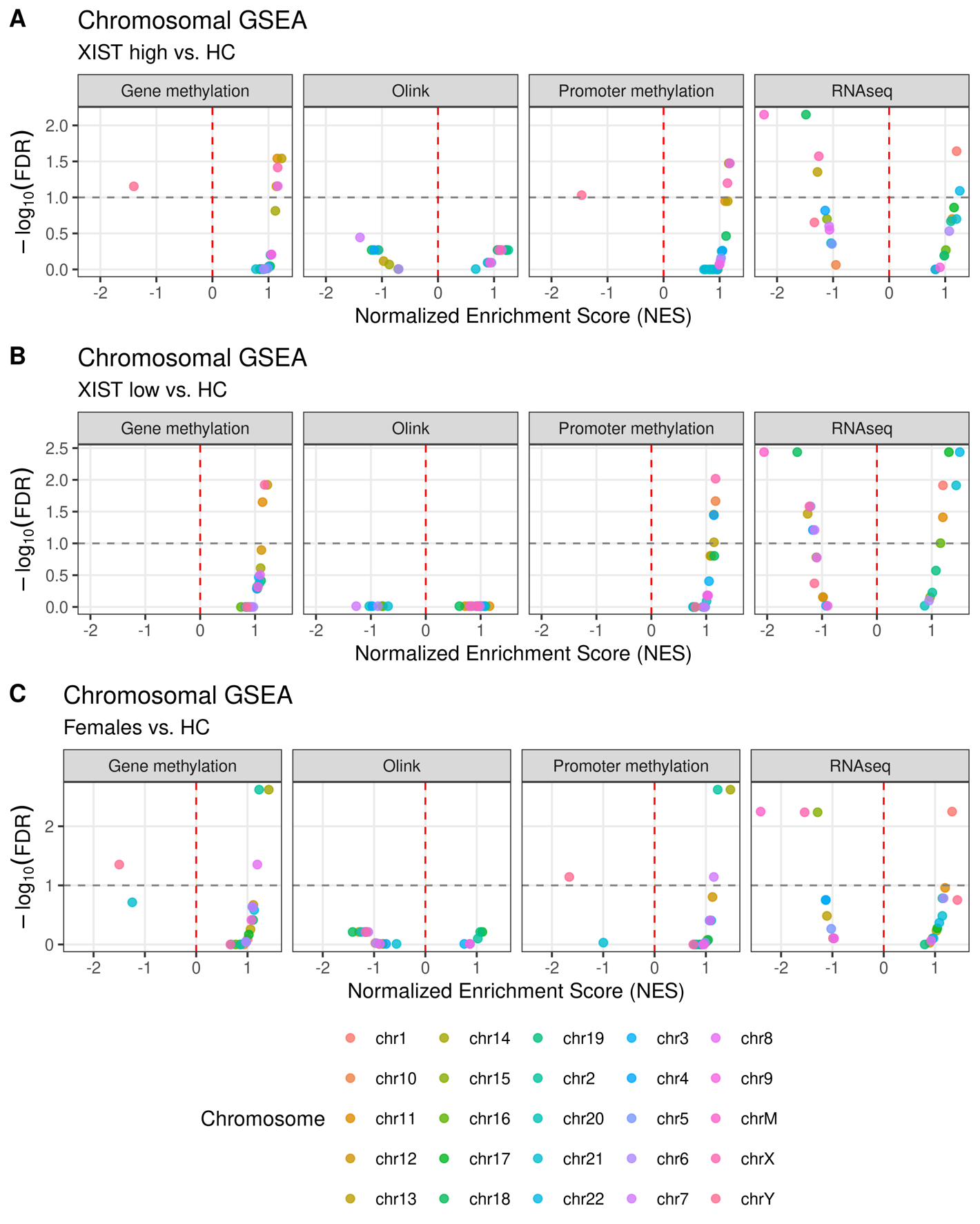


**Supplementary Figure 3. Chromosomal gene set enrichment analysis (GSEA).**  Volcano plots illustrating chromosomal GSEA results for each modality: **(A)** XIST high males vs. healthy control males, **(B)** XIST low males vs. healthy control males, **(C)** Females vs. healthy controls. Dot colors correspond to individual chromosomes. Dots above the dashed grey line represent significant enrichment (FDR ≤ 0.1). Dots to the right of the dashed red line indicate positive enrichment, while those to the left of the dashed red line indicate negative enrichment. For methylation data, note that positive enrichment corresponds to increase methylation which is typically associated with gene silencing.

**Supplementary Figure 4. Benchmarking for immune cell deconvolution.**  We tested 4 different reference panels (ABIS_S0, ABIS_S1, ABIS_S2, and ABIS_S3) in combination with 7 deconvolution algorithms: **A)** dtangle **B)** non-negative least squares regression (nnls), **C)** ordinary least squares (ols) **D)** quadratic programming without constraints (qprog), **E)** quadratic programming non-negative and sun-to-one constraints (qprogwc), **F)** robust linear regression (rls), and **G)** support vector regression (svr). These correlation heatmaps show concordance (Pearson correlation) between the estimated immune cell types from each deconvolution (y-axis) with the known cell type proportions measured via flow cytometry (x-axis)

**Supplementary Table 12:** Correlation of type 1 IFN signature and XIST levels in males and females with SLE

|  | **Females** | | **Males** | |
| --- | --- | --- | --- | --- |
|  | **Correlation (r)** | **P-value** | **Correlation (r)** | **P-value** |
| **MX1** | -0.30 | 8.31e-16 | 0.00 | 0.99 |
| **OAS3** | -0.19 | 3.35e-07 | 0.06 | 0.70 |
| **DDX60** | -0.01 | 7.75e-01 | 0.11 | 0.50 |
| **LY6E** | -0.18 | 2.18e-06 | 0.13 | 0.43 |
| **USP18** | -0.08 | 0.03 | 0.15 | 0.35 |
| **IFI44** | -0.03 | 0.44 | 0.17 | 0.30 |
| **IFN signature** | -0.16 | 3.56e-05 | 0.07 | 0.68 |

**Supplementary Table 1** – Promoter methylation

1. Differentially methylated promoters in females (SLE vs healthy controls)
2. Differentially methylated promoters in males (SLE vs healthy controls)
3. Gene Set Enrichment Analysis results (GSEA) in females
4. Gene Set Enrichment Analysis results (GSEA) in males

**Supplementary Table 2** – Gene methylation

1. Differentially methylated genes in females (SLE vs healthy controls)
2. Differentially methylated geness in males (SLE vs healthy controls)
3. Gene Set Enrichment Analysis results (GSEA) in females
4. Gene Set Enrichment Analysis results (GSEA) in males

**Supplementary Table 3** – RNAseq

1. Differentially expressed genes in females (SLE vs healthy controls)
2. Differentially expressed genes in males (SLE vs healthy controls)
3. Hypergeometric enrichment analysis results of commonly upregulated genes
4. Hypergeometric enrichment analysis results of commonly downregulated genes

**Supplementary Table 4** – Olink

1. Differentially expressed proteins in females (SLE vs healthy controls)
2. Differentially expressed proteins in males (SLE vs healthy controls)
3. Gene Set Enrichment Analysis results (GSEA) in females
4. Gene Set Enrichment Analysis results (GSEA) in males

**Supplementary Table 5** – Significant sex:disease interactions

1. Olink
2. RNA-seq
3. Gene Methylation
4. Promoter Methylation

**Supplementary Table 6** – Chromosomal Gene Set Enrichment Analysis (GSEA) results

1. Custom gene set list – RNAseq
2. Custom gene set list – Olink
3. Custom gene set list – Gene methylation
4. Custom gene set list – Promoter methylation
5. GSEA results

**Supplementary Table 7** – X-chromosome inactivation (XCI) genes and their changes in XIST-high and XIST-low males compared to male healthy controls

**Supplementary Table 8** – DEA by XIST subgroup: XIST-high vs HC, XIST-low vs HC, and XIST-high vs XIST-low

1. RNA-seq
2. Olink

**Supplementary Table 9** – DEA by XIST subgroup: XIST-high vs HC, XIST-low vs HC, and XIST-high vs XIST-low

1. Promoter Methylation
2. Gene Methylation

**Supplementary Table 10** – Comparison of clinical parameters between XIST high vs XIST low male SLE patients

1. 95 numerical clinical parameters
2. 31 categorical clinical parameters

**Supplementary Table 11** – Clinical correlates (spearman) with XIST expression

1. All SLE patients (males + females)
2. Male SLE only
